# Spermatogenesis is normal in *Tex33* knockout mice

**DOI:** 10.7717/peerj.9629

**Published:** 2020-07-29

**Authors:** Zhendong Zhu, Xin Zhang, Wentao Zeng, Shuqin Zhao, Jianli Zhou, Zuomin Zhou, Mingxi Liu

**Affiliations:** 1 Department of Histology and Embryology, Nanjing Medical University, Nanjing, China; 2 Animal Core Facility of Nanjing Medical University, Nanjing Medical University, Nanjing, China

**Keywords:** *Tex33*, Spermatogenesis, Mouse, NIRH and SYT domain, Male infertility

## Abstract

Testis expressed gene 33 (*Tex33*) is a recently reported testis-specific gene and it is evolutionarily conserved in vertebrates. The *Tex33* expression is found in cytoplasm of round spermatids in Mus musculus. However, the in vivo function of *Tex33* remains unknown. In this study, we made a 62bp in frame deletion on Exon2 of *Tex33* gene by CRISPR/Cas9 in C57B/L6 mouse, which cause frame shift mutation of *Tex33* gene. *Tex33*^-/-^adult male were fertile, and there is no significant change on litter size compared with male wildtype (*Tex33*^+/+^) adult. Besides, no overt differences were found in testis/body weight ratios, testicular/epididymal tissue morphology, sperm counts, sperm morphology and spermatozoa motility in adult *Tex33^-/-^*male mice (*N* = 3), in comparison with *Tex33*^+/+^ adult (*N* = 3). TUNEL assay also indicates the germ cells apoptosis ratio was not significantly changed in adult *Tex33^-/-^* adult male mouse testis (*N* = 3), compared with adult *Tex33^+/+^* male (*N* = 3). Importantly, the first wave of elongating spermatids formation happens in 5w old mice. We find that the first wave of spermiogenesis is not disrupted in both 5-week-old *Tex33^+/+^* and *Tex33*^-/-^male mouse testes and three hallmarks of spermatogenesis, PLZF,*γ*-H2AX and TNP1, are all detectable in seminiferous tubule. All results indicate that *Tex33* is a redundant gene to spermatogenesis. This study can help other researchers avoid repetitive works on redundant genes.

## Introduction

Spermatogenesis is a complex procedure and produces functional haploid sperm from diploid germ cells in various species (Hess & Franca, 2009). It involves three major steps: (1) the division and proliferation of spermatogonia via mitosis; (2) the meiosis takes part in the segregation of homologous chromosomes; (3) spermiogenesis makes round spermatid transformed into the elongated spermatids, which later detach from seminiferous epithelium (De Kretser et al., 1998). The germ cells express a large number of testis-enriched genes, many of these genes may play roles in the procedures of mitosis, meiosis and spermiogenesis (Eddy, 2002).

More than 2,300 genes are testicular predominantly expressed genes in *Mus Musculus* (Schultz, Hamra & Garbers, 2003). Until now, hundreds of genes are reported necessary to mouse spermatogenesis. However, many genes are redundant to male fertility (Felipe-Medina et al., 2019; Hasegawa, Okamura & Saga, 2012; Huang, Rivas & Agoulnik, 2012; Nie, Dai & Luo, 2020; Sacher et al., 2007). Recently, Lu et al. (2019) report that 30 evolutionary conserved testis-enriched mutant genes are inessential to spermatogenesis in *Mus Musculus,* which suggests the possibility that some testis-specific genes may be redundant to germ cells. Though the discovery of testis-enriched genes which are in relate to spermatogenesis have their significance to explore the mechanism of mitosis, meiosis and spermiogenesis, many testis-specific redundant genes are still valuable for researchers. Reports of these redundant genes can avoid wasting times and resources into the gene knocked-out mouse with fertile phenotype in the study of male reproduction. There are still numerous testis-specific genes the in vivo function of which are unknown. In order to discover the in vivo functions of these testicular enriched genes, the CRISPR/Cas9 gene knock-out strategy is a commonly used method (Khan et al., 2018; Miyata et al., 2016). The advantage of CRISPR/Cas9 edited testis-enriched gene KO mouse model is obvious. It does not need the complex procedure of conditional knock-out because the male gonad dysfunction is not fatal to the rodent animal, and the phenotype of fertility dysfunction is easy to find (Miyata et al., 2016). Moreover, CRISPR/Cas9 is an affordable and quicker way to create gene mutations in a shorter time (Ma, Zhang & Huang, 2014).

*Tex33* is a recently discovered evolutionarily conserved gene present in vertebrates, which is initially expressed in the cytoplasm of round spermatids, and is diminished in elongated spermatids (Kwon et al., 2017). Until now, *Tex33* knockout animal model has not been reported. Therefore, we generated a 62 bp in frame shift mutation on the second exon of *Tex33* gene in C57B/L6 mouse via CRISPR/Cas9 system to discover the reproductive phenotype of *Tex33*. Our findings suggest that testis-enriched *Tex33* is dispensable to spermatogenesis.

## Material and Methods

### The in-frame deletion of Tex33 gene generated via CRISPR/Cas9

The experimental animal usage protocol had been approved by the Institution of Animal Care and Use Committee (Approval: IACUC1811001-2, Nanjing Medical University, China). In this study, all *Tex33*^+∕+^, *Tex33*^+∕−^ and *Tex33*^−∕−^ mouse were housed and all experiments were implemented in the line with the guidance from the Institution of Animal Care and Use Committee (Nanjing Medical University, China). The generation of Tex33 knock-out mice were based on CRISPR/Cas9 techniques. Besides, both the mRNA of *Cas9* and the sgRNA were prepared with our previous study (Zhang et al., 2017). In short, the circular *Cas9* plasmid (Addgene, Watertown, MA, USA) was digested by endo-nuclease: *AgeI.* The linearized plasmid is then purified by MinElute PCR Purification Kit (Qiagen, Duesseldorf, Germany). Next, the linear *in vitro* transcribed *Cas9* was produced via mMESSAGE mMACHINE T7 Ultra Kit (Amibion, Austin, TX, USA), that it‘s then treated by RNeasy Mini Kit (Qiagen, Duesseldorf, Germany) for purification. sgRNA were designed on the second exon on Tex33 gene. The target DNA sequence with PAM were: 5′-GGTCTAGGTCGAGCTCTCTACGG-3′ and 5′-GGGAGGAAGGCCAAGACTCCAGG-3′. The circular sgRNA template plasmid was cut by restriction endonuclease *DraI* for linearization and then the linearized sgRNA is treated by MinElute PCR Purification Kit (Qiagen, Duesseldorf, Germany). The sgRNA is *in vitro* transcribed by MEGA shortscript Kit (Ambion, Austin, TX, USA). Besides, sgRNA product was purified by using MEGA clear Kit (Ambion, Austin, TX, USA) followed by producer’s protocols. Finally, we spontaneously inject both *in vitro* transcribed products, Cas9 and sgRNA, into the fertilized super-ovulated wild-type C57B/L6 female mouse zygotes, mated by wild-type C57B/L6 male mouse.

### Genotyping

Mouse genome DNA is collected from mice tail specimen and the detect the deletion of *Tex33* gene by PCR and nucleic acid electrophoresis. The founders (F0) of gene edited mouse were identified by two detection primers: Primer F 5′-GTACAACCACGTTGACAAGG-3′ and Primer R 5′-CCTCATTAAAAGCCTCTAAG-3′ and PCR (Fast-Taq master mix, Vazyme). The PCR product is subcloned to T-vector (pMD19-T, Takara) and the subcloned T-vectors were undergone the Sanger sequencing. Detected nucleotide sequence length (nt) from the wild-type allele was 502 bp while the mutant allele was 440 bp. The target founder (F0) was mated with wild-type (*Tex33*^+∕+^) C57BL/6 adult mice in order to avoid the possibility of off-targets effect and then produce pure heterozygous mice. *Tex33*^−∕−^ mice genome DNA strand were sequenced via Sanger DNA sequencing. The sequencing result is plotted by Snapgene (version 1.1.3).

### Fertility test

Adult *Tex33*^+∕+^ and *Tex33*^−∕−^ male mice undergo fertility tests. In this study, each male in experimental group and control group is being mated with 3 adult *Tex33*^+∕+^ C57BL/6 female mice. *Tex33*^−∕−^ isset as experiment group while *Tex33*^−∕−^ is set as control group. Every morning, the presence of a vaginal plug from female mice was checked. In each litter, the birth date and pups’ number were recorded and undergone statistic.

### Western blot assay

The adult mice testicular parenchyma protein from all genotypes were extracted by using protein lysis buffer (75 mM NaCl, 50 mM Tris-HCl, 8 M urea, pH 8.2) with 1 × Complete™ EDTA-free Protease Inhibitor Cocktail (Roche, Basel, Switzerland). The adult male mice testicular protein from all genotypes were separated via dodecyl sulfate sodium salt-polyacrylamide gel electrophoresis (SDS-PAGE) electrophoresis. The sample is transferred onto methanol-activated polyvinylidene difluoride (PVDF) membrane. Later, PVDF membranes were blocked by TBST (150 mM NaCl, 20 mM Tris, 0.1% Tween 20) with 5% non-fat milk for 2 h at room temperature. Later, the blocked PVDF membrane was incubated 4 °C for 12 h, with detectable primary antibody anti-Tex33 (ab121241; Abcam Biotechnology, China) at a 1:250 dilution. Besides, the PVDF membrane is incubated by β-Tubulin (ac015, ABclonal, Wuhan, China) at the dilution at 1:1000, which is set as internal control. The PVDF membranes were washed by TBST for three times and each lasts 5 min. Then, the membrane was under incubation with secondary detection antibodies at the 1:1000 dilution for 2 h at room temperature. The luminescent signals of the target proteins were detected by High-sig ECL Western Blotting Substrate (Tanon, Shanghai, China).

### Animal histological analysis

The testis and epididymis were extracted from at least three mice from *Tex33*^+∕+^, *Tex33*^+∕−^ and *Tex33*^−∕−^ male respectively. The male gonad and epididymal tissues were fixed by the usage of modified Davidson’s fluid (MDF) for 24 h. The organs and tissues were then got stored in 70% ethanol. Stored testicular and epididymal tissues were later dehydrated by a set of graded dilution ethanol (80%, 90%, 100%). The tissues are finally embedded in paraffin. Testis and epididymis tissue sections (5-mm thick) were flatten on water (37 °C) and mounted to glass slides. After deparaffinized by dimethylbenzene, the tissue was stained by periodic acid Schiff (PAS) for histological analysis.

Seminiferous tubule diameter and epithelium height (thickness) were recorded by Zeiss Axio Skop Plus 2 (Carl Zeiss AG, Jena, Germany), from each section. 20 of round or nearly round testis seminiferous tubules were randomly chosen in adult *Tex33* ^+∕+^ C57BL/6 mice (*N* = 3) and adult *Tex33*^−∕−^ C57BL/6 mice (*N* = 3). The tubules diameter and epithelium height were measured by AxioVision Rel 4.8 (Carl Zeiss AG, Jena, Germany). Epithelium height is the vertical length from epithelium thickest part apex to the basal membrane of seminiferous tubules and the tubular diameter is the distance between the farthest two points of the tubule. Besides, 3 of round or nearly round Stage7∼ Stage8 seminiferous tubules were randomly chosen in one testis slice of *Tex33*^+∕+^ male adult C57BL/6 mice (*N* = 3) and *Tex33*^−∕−^ male adult C57BL/6 mice (*N* = 3). The total germ cells per tubule were counted and cataloged. The germ cells were counted via software (Adobe Photoshop). Apoptotic cells in the seminiferous tubule from the adult testis were detected by the usage of TUNEL assay (Vazyme, Nanjing, China), in line with the producer’s guidelines.

### Immunofluorescence

Testicular tissue and sperm specimen underwent deparaffinization, rehydration, and antigen repair (immerged in in sodium citrate buffer, heated for 15 min). The tissue and specimen were blocked by 1x phosphate buffered saline (PBS) with 5% bovine serum albumin at room temperature for 2 h, followed with primary antibodies incubation at 4 °C overnight (See in Table S18). PBST (1xPBS with 0.05% Tween 20) washes testicular and sperm specimen for 5 min, three times. The tissue specimen was incubated with secondary antibodies for 2 h at room temperature. Finally, the nucleus was stained by Hoechst 33342 (Invitrogen, Carlsbad, CA, USA) for 5 min. The specimen is finally mounted. Immunofluorescence Images were captured and observed by LSM800 confocal microscope system (Carl Zeiss AG, Jena, Germany).

### Computer-assisted sperm analysis (CASA)

Sperm from the adult male were collected by making a cross incisions throughout the epididymis cauda. The epididymal spermatozoa was extruded and suspended in human tubal fluid culture medium (In Vitro Care, Frederick, MD, USA). 10 µL of epididymis sperm suspension fluid were used for CASA (Hamilton-Thorne Research, Inc, Beverly, MA, USA). The ratio of motile spermatozoon, progressive motile spermatozoon percentage and the spermatozoon concentration (M/ml) in *Tex33*^−∕−^ (experiment group) and *Tex33*^+∕+^ (control group) adult male mice were undergone recorded and analyzed.

### Statistical analysis

The procedures of experiment are all repeated at least 3 times. Besides, one-way analysis of variance (one-way ANOVA) and unpaired two-tailed *t*-tests were two methods to analyze the differences between experimental group and control group. It‘s considered that *P* < 0.05 indicates there exists a statistically significant difference between experimental group and control group. All data indicate the mean ± the standard error of the mean. Two software, Microsoft Excel, and GraphPad Prism 8.0 are used for statistical analysis.

## Results

### *Tex33* CRISPR/Cas9 knocked out C57B/L6 mouse is successfully generated

**Figure 1:**
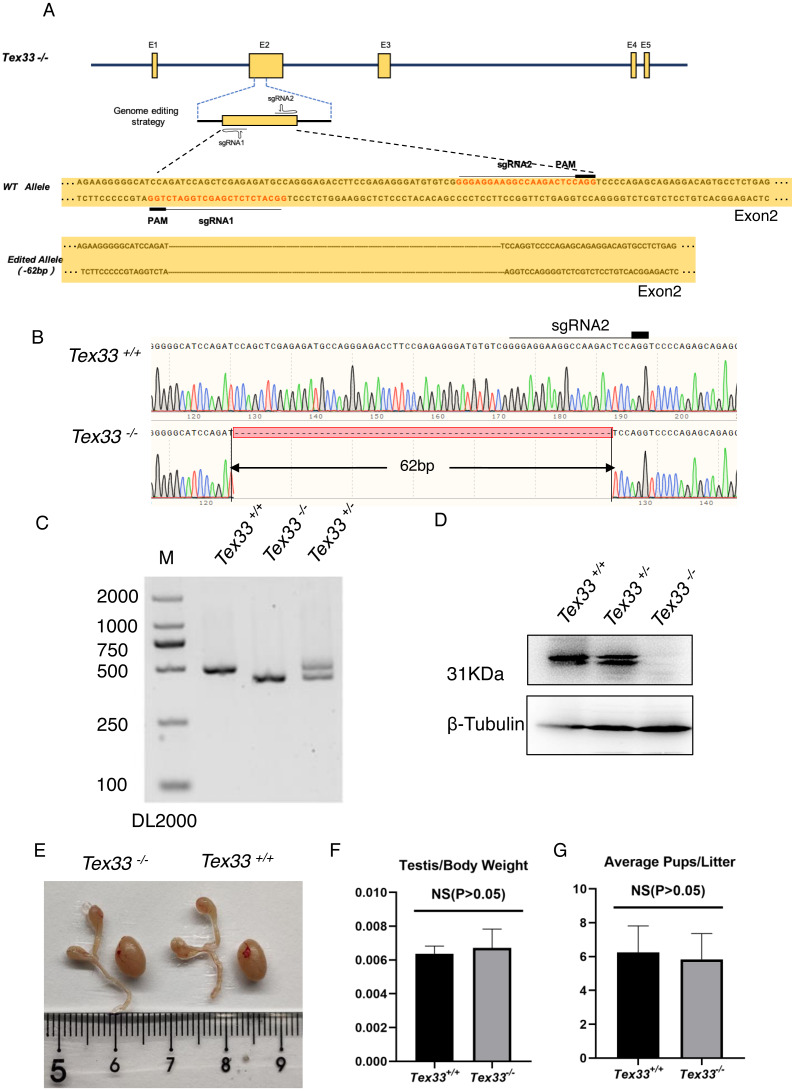
62 bp in-frame deletion on Exon2 of *Tex33*^−∕−^ is generated by CRISPR/Cas9. (A) Schematic diagram of CRISPR/Cas9 targeting strategy; the sgRNAs were designed within exon 2 of *Tex33*; (B) A 62-bp deletion on exon 2 of *Tex33* was detected in F2 *Tex33*^−∕−^ mice by sanger sequencing (C) Genotyping of *Tex33*^+∕+^, *Tex33*^+∕−^ and *Tex33*^−∕−^ mice. (D) TEX33 is not detected in *Tex33*^−∕−^ male mouse testis by Western blot. (E) Testis and epididymis from wild-type and * Tex33*^−∕−^ adult mice. (F) Average Testis weight/body weight of adult *Tex33*^−∕−^ male,*n*= 3,*P*> 0.05. (G) Average pups per litter of adult wild-type male and *Tex33*^−∕−^ male mice, *n* = 3, *P* > 0.05.

**Figure 2:**
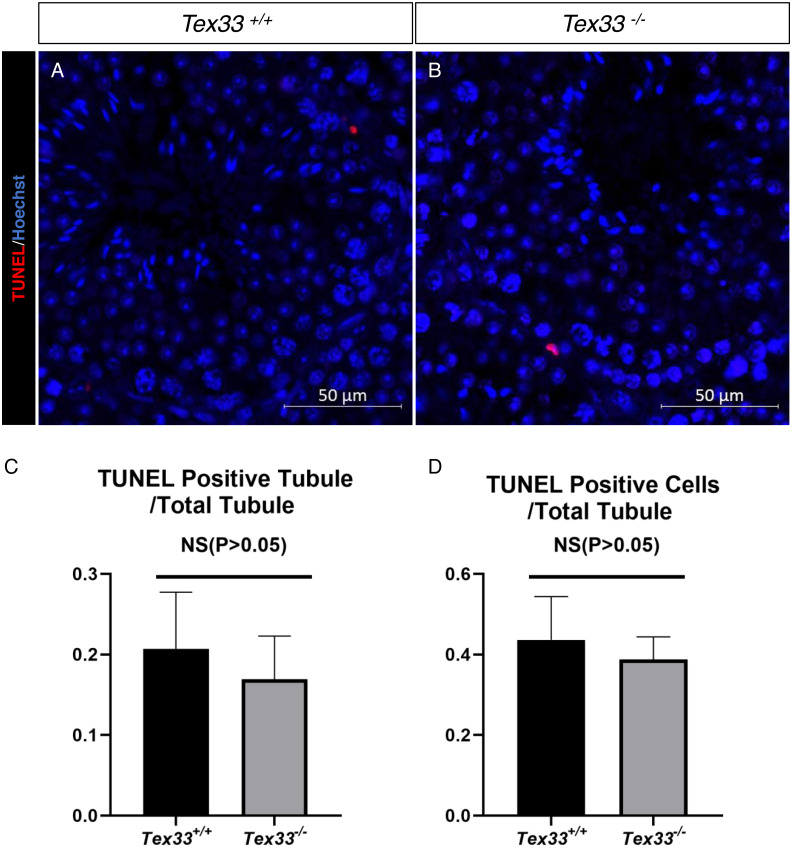
Apoptotic cells are not increased in adult *Tex33*^−∕−^ male testes. TUNEL assay of (A) wild-type and (B) *Tex33*^−∕−^ testes; (C) average TUNEL positive apoptotic tubule counts; (D) average TUNEL positive apoptotic cells counts,*n*= 3,*P*> 0.05.

To find the in vivo function of the *Tex33* gene, we generated a in frame deletion in the second exon of *Tex33* gene in super-ovulated fertilized eggs by using the CRISPR/Cas9 (Fig. 1A). Compared with the wild-type control groups, we examined a 62bp loss on the second exon of Tex33 gene in F2 *Tex33*^−∕−^ mouse by sanger sequencing and PCR genotyping (Figs. 1B–1C). Due to the 62bp loss will cause frame shift mutation on *Tex33*, we find that TEX33 is not detectable in adult F2 *Tex33*^−∕−^ mouse testis by Western blot, whereas the F2 *Tex33*^+∕−^ mice can still express high level of TEX33 in testis (Fig. 1D, Fig. S1). The epididymis and testis morphology are normal in adult *Tex33*^−∕−^ mice (Fig. 1E). Testis weight of *Tex33*^−∕−^ adult mouse (*N* = 3) is not significantly changed, compared with male wildtype control group (*N* = 3) (Fig. 1F). Testis and epididymis morphology are also normal in adult *Tex33*^+∕−^ male mice, compared to the adult wildtype male testis (Fig. S2A). We find the *Tex33*^−∕−^ male adult mouse (*N* = 3) are fertile and the litter size is not significantly changed in contrast to adult male wild-type control group (*N* = 3) (Fig. 1G). Meanwhile, the germ cell apoptotic ratio is also not significantly changed in adult *Tex33*^−∕−^ males, in contrast to wild type control groups (*N* = 3) (Figs. 2A–2D).

**Figure 3:**
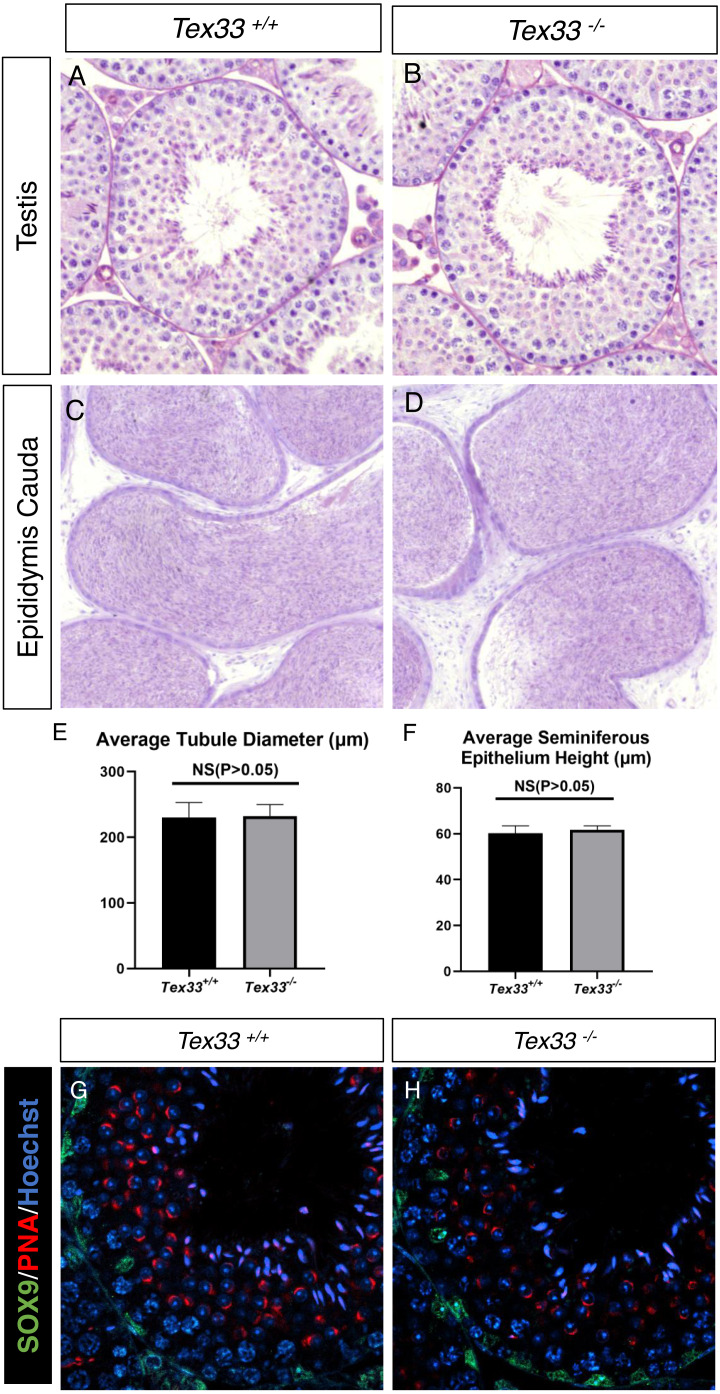
Spermatogenesis is normal in*Tex33*^−∕−^ mice. Sections of periodic acid Schiff-stained testis from adult (A) wild-type and (B)* Tex33*^−∕−^ mice; Sections of hematoxylin and eosin-stained cauda epididymis from adult (C) wild-type and (D) *Tex33*^−∕−^ mice. (E) Average tubule diameter of adult wild-type and *Tex33*^−∕−^ male mice, *n* = 3, *P* > 0.05. (F) Average seminiferous epithelium height of adult wild-type and *Tex33*^−∕−^ male mice,n= 3,*P*> 0.05. Sertoli cells (SOX9) and Spermatids (PNA) is comparable in adult (G) wild-type and (H) *Tex33*^−∕−^ male mice testis.

**Figure 4:**
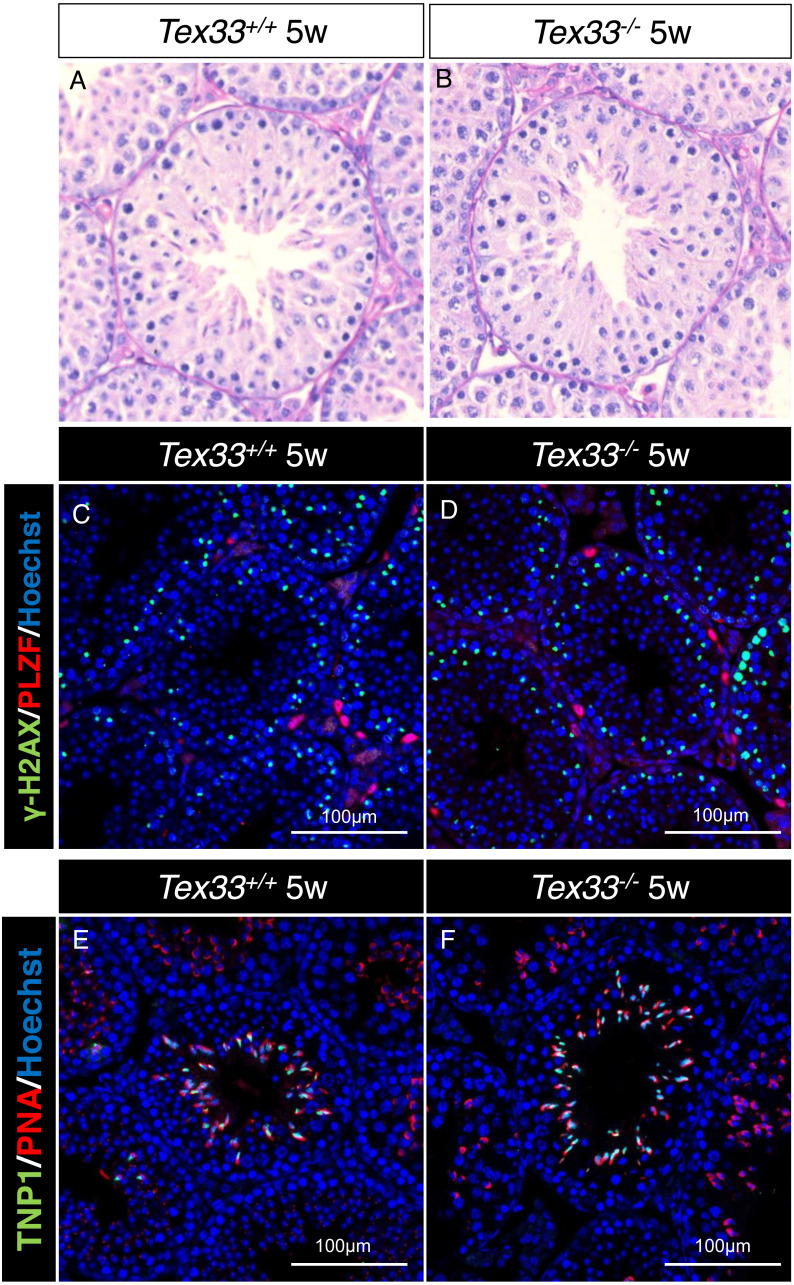
First wave of spermatogenesis is not disrupted in 5w *Tex33*^−∕−^ mice. Sections of periodic acid Schiff-stained testis from 5w (A) wild-type and (B) * Tex33*^−∕−^ mice. The spermatogonia (PLZF), spermatocytes (*γ*-H2AX) are comparable in testis sections from both 5w (C) wild-type and (D) *Tex33*^−∕−^ mice; Spermatids (PNA) and elongated spermatids (TNP1) are comparable in testis sections from both 5w (E) wild-type and (F) * Tex33*^−∕−^ mice.

### Spermatogenesis is normal in *Tex33*^−∕−^ male mouse

We observe the histological change on F2 adult *Tex33*^−∕−^ and *Tex33*^+∕−^male mouse (*N* = 3), and adult *Tex33*^+∕+^ F2 male is set as control groups (*N* = 3). Both spermatids and spermatocytes exist in seminiferous tubule on F2 *Tex33*^+∕+^, *Tex33*^+∕−^ and *Tex33*^−∕−^ male mouse testis (Figs. 3A–3B, Figs. S2B–S2C). Besides, vast spermatids cells are found in epididymis cauda of F2 adult *Tex33*^+∕+^ and*Tex33*^−∕−^ male mouse (Figs. 3C–3D). The tubular diameter and seminiferous epithelium height are not significantly changed in *Tex33*^−∕−^ mouse (*N* = 3), in contrast to control group (*N* = 3) (Figs. 3E–3F). Moreover, the spermatogenic phase is not disrupted in adult *Tex33*^−∕−^ male (Fig. S2D) and the average proportion of germ cells in tubule (spermatogonia cells, spermatocytes, round spermatid and long spermatid) is not significantly changed in adult *Tex33*^−∕−^ male testis (Fig. S2E–S2H). SOX9 is a Sertoli-cell specific protein and it‘s playing an essential role in spermatogenesis (Daigle, Roumaud & Martin, 2015). SOX9 is detectable and comparable in both *Tex33*^+∕+^ and *Tex33*^−∕−^ adult mouse (Figs. 3G–3H). In order to find whether TEX33 deletion would affect the first wave of spermatogenesis, we extract the testes from F2 5w *Tex33*^+∕+^ and 5w *Tex33*^−∕−^male mouse. The first wave of elongating spermatids formation happens in 5w mice (Bellve et al., 1977). We can see elongated spermatids are existed in seminiferous tubule on both 5w *Tex33*^+∕+^ and *Tex33*^−∕−^ male (Figs. 4A–4B). PLZF, *γ*-H2AX and TNP1 exclusively express in proliferating spermatogonia cells, primary spermatocytes during meiosis and elongating spermatids respectively, and they are also the hallmarks of spermatogenesis (Costoya et al., 2004; Fernandez-Capetillo et al., 2003; Meistrich & Hess, 2013; Yu et al., 2000). All of these hallmarks are detectable in 5w *Tex33*^+∕+^ and *Tex33*^−∕−^mouse testis (Figs. 4C–4F).

### Semen quality is normal in adult *Tex33*^−∕−^ male mouse

**Figure 5:**
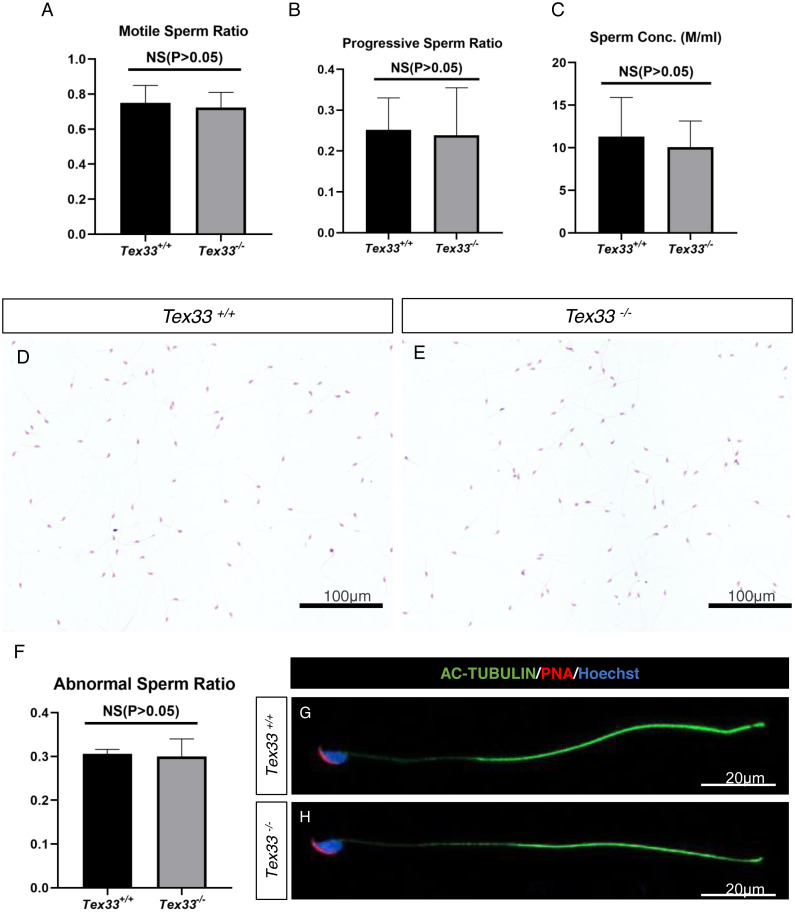
Spermatozoa appears normal in *Tex33*^−∕−^ mice. (A) Average ratio of motile sperm and (B) progressive sperm from adult wild-type and*Tex33*^−∕−^ mice, *n*= 3,*P*> 0.05; (C) Normal epididymal sperm concentration from adult wild-type and *Tex33* ^−∕−^ mice, *n*= 3,*P*> 0.05. Hematoxylin and eosin-stained spermatozoa fromadult (D) wild-type and (E) *Tex33*^−∕−^ and wild-type mice. (F) Cauda epididymal sperm abnormality ratio from adult wild-type and*Tex33*^−∕−^ mice,*n*= 3,*P*> 0.05. Fluorescence detection of AC-TUBULIN, PNA from adult (G) wild-type and(H) *Tex33*^−∕−^ spermatozoa.

To evaluate semen quality of *Tex33*^−∕−^ adult mouse, computer assisted spermatozoon analysis (CASA) is used to statistic the sperm motility ratio, progressive sperm ratio and sperm amount. We find that there‘s no significant change on semen quality on *Tex33*^−∕−^male mouse (*N* = 3), compared with *Tex33*^+∕+^ male (*N* = 3) (Figs. 5A–5C). No significant change on semen quality is also found in adult *Tex33*^+∕−^male (Figs. S3A–S3C). The spermatozoon is extracted from the epididymis cauda and got HE stained (Figs. 5D–5E). The morphological abnormal sperm ratio is not significantly changed in *Tex33*^−∕−^ male adult mouse (*N* = 3), in comparison with *Tex33*^+∕+^male (*N* = 3) (Fig. 5F). IF results also indicate that the sperm flagellar is intact in *Tex33*^−∕−^ male adult mouse, same as wild type mouse (Figs. 5G–5H).

## Discussion

Nowadays, various genes and non-coding transcripts are reported got involved in the regulation of sperm formation. Previous studies have identified several sperm-specific proteins and their related genes, such as the sperm calcium channel protein CatSper family gene, potassium ion channel protein Slo3, and sperm proton transporter sNHE etc (Miller et al., 2015). The inactivation of these proteins and their related genes incurs defect in sperm motility (Qi et al., 2007; Wang et al., 2007; Zeng et al., 2011). Non coding transcripts also got involved in the spermatogenesis. piRNA is a class of small non-coding RNA and in relate to PIWI proteins, which vitally takes part in retrotransposon silencing and epigenetic controls (Beyret, Liu & Lin, 2012). piRNA biogenesis disruption cause spermatogenesis failure. *Pnldc1* takes part in piRNA trimming and the deletion of *Pnldc1* would cause severe spermatogenesis arrest with abnormal activation of retrotransposons LINE1 (Ding et al., 2017; Zhang et al., 2017). Importantly, these genes are all testis-enriched. In order to find out more genes with potential roles to spermatogenesis, the discovery of in vivo functions of testis-enriched genes is a promising job. It would unveils the mechanism of spermatogonium cells stemness, meiosis, spermiogenesis and so on (Li, Lee & Zhang, 2005). Even though numerous testis-enriched gene KO mouse models have validated male fertility impairment (e.g., *Tcte1*, *Fbxo47* and *Shcbp1l* etc.), knocking out of some testis-enriched genes still incurs fertile phenotype (e.g., *Fank1*, *Tmco5*, *Smim23* and *March11* etc.) (Castaneda et al., 2017; Hua et al., 2019; Liu et al., 2014; Miyata et al., 2016; Zhang et al., 2019).

*Tex33* is an evolutionary conserved and testis-specific gene. It is transcribed in round spermatids and it does not exist in testicular elongated spermatids and epididymal sperm(Kwon et al., 2017). In this study, we generated a 62bp in frame deletion on Exon2 of *Tex33* in C57B/L6 mouse model by using the CRISPR/Cas9 system. *Tex33*^−∕−^male adult C57B/L6 male mouse are fertile and there‘s no morphological and histological changes in gonads and gametes, compared with the *Tex33*^+∕+^ male control group. The loss of Tex33 does also not affect the first wave of spermatogenesis. We assure vthat *Tex33* is dispensable to male fertility. The fertile phenotype of *Tex33*^−∕−^ male mouse can be explained by gene redundancy. Redundancy is the phenomenon that two or more gene products are performing the same or overlapping function in a given physiogical or cellular context and the inactivation of one of the redundant genes will be compensated by other genes with similar function and has little or no effect on the organism itself (Nowak et al., 1997). Till now, many testis-specific-genes are reported the redundant gene and the knocking out of these genes do not impair male fertility in mouse models (Iwamori et al., 2011; Lu et al., 2019; Ozturk et al., 2014). Due to the lack of evidence of domains or sequence motifs in *Tex33*, it‘s still hard to explain which gene replaces the Tex33 in vivo yet. Recent study implies redundant testis-specific genes may not be idle or useless to the male, because the initiation of vast gene expression can prevent germ cell’s DNA mutation via transcription-coupled repair (TCR), in order to avoid hereditary of wrong DNA message to the offspring(Xia et al., 2020). Our study suggests that a testis-predominantly expressed genes Tex33 is not necessary to spermatogenesis and male fertility, we should focus on genes that are indispensable to male fertility.

##  Supplemental Information

10.7717/peerj.9629/supp-1 Table S1 Testis/Body weight of male adult *Tex33*^+∕+^ and *Tex33*^−∕−^ mice

10.7717/peerj.9629/supp-2 Table S2 Average pups per litter of *Tex33*^+∕+^ and *Tex33*^−∕−^ male mice

10.7717/peerj.9629/supp-3 Table S3 TUNEL positive tubule ratio of *Tex33*^+∕+^ and *Tex33*^−∕−^ adult male mice

10.7717/peerj.9629/supp-4 Supplemental Information 4 TUNEL positive cell ratio of *Tex33*^+∕+^ and *Tex33*^+∕+^ adult male mice

10.7717/peerj.9629/supp-5 Table S5 Average seminiferous tubule diameter of *Tex33*^+∕+^ and *Tex33*^−∕−^ adult male mice (µm)

10.7717/peerj.9629/supp-6 Table S6 Average seminiferous epithelium height of *Tex33*^+∕+^ and Tex33^−∕−^ adult male mice

10.7717/peerj.9629/supp-7 Table S7 Motile sperm ratio of adult *Tex33*^+∕+^ and *Tex33*^−∕−^ male mice

10.7717/peerj.9629/supp-8 Table S8 Progressive sperm ratio of adult *Tex33*^+∕+^ and *Tex33*^−∕−^ male mice

10.7717/peerj.9629/supp-9 Table S9 Sperm concentration of adult *Tex33*^+∕+^ and *Tex33*^−∕−^ male mice (M/ml)

10.7717/peerj.9629/supp-10 Table S10 Sperm abnormality ratio of *Tex33*^+∕+^ and *Tex33*^−∕−^ male adult mice

10.7717/peerj.9629/supp-11 Table S11 Average spermatogonia cell proportion in male adult *Tex33*^+∕+^ and *Tex33*^−∕−^ mice.

10.7717/peerj.9629/supp-12 Table S12 Average spermatocyte proportion in male adult *Tex33*^+∕+^ and *Tex33*^−∕−^ mice

10.7717/peerj.9629/supp-13 Table S13 Average round spermatid proportion in male adult *Tex33*^+∕+^ and *Tex33*^−∕−^ mice

10.7717/peerj.9629/supp-14 Table S14 Average long spermatid proportion in male adult *Tex33*^+∕+^ and *Tex33*^−∕−^ mice.

10.7717/peerj.9629/supp-15 Table S15 Motile sperm ratio in male adult *Tex33*^+∕+^ and *Tex33*^+∕−^ mice (%)

10.7717/peerj.9629/supp-16 Table S16 Progressive sperm ratio in male adult *Tex33*^+∕+^ and *Tex33*^+∕−^ mice (%)

10.7717/peerj.9629/supp-17 Table S17 Sperm concentration in male adult *Tex33*^+∕+^ and *Tex33*^+∕−^ mice (M/ml)

10.7717/peerj.9629/supp-18 Table S18 Antibodies List

10.7717/peerj.9629/supp-19 Figure S1 The TEX33 detection in *Tex33*^+∕+^, * Tex33*^+∕−^ and *Tex33*^−∕−^ testis of adult male mouse by Western blot TEX33 is detected in *Tex33*^+∕+^ and * Tex33*^+∕−^ adult male testis, and beta-Tubulin is set as internal control

10.7717/peerj.9629/supp-20 Figure S20 Normal Spermatogenesis in *Tex33*^+∕−^ and *Tex33*^−∕−^ seminiferous tubule (A) Testis and epididymis from adult wild-type and * Tex33*^+∕−^ adult mice. Sections of periodic acid Schiff-stained testis from adult (B) wild-type and (C) *Tex33*^+∕−^ mice; (D) Compared with the wild-type mouse, the adult *Tex33*^−∕−^ mouse have no spermatogenic abruption and failure in Sections of periodic acid Schiff-stained testis. (E) Average Spermatogonia cells ratio in adult wild-type and *Tex33*^−∕−^ mice, *n* = 3, *P* > 0.05. (F) Average Spermatocytes ratio in adult wild-type and *Tex33*^−∕−^ mice, *n* = 3, *P* > 0.05. (G) Average round spermatids ratio in adult wild-type and *Tex33*^−∕−^ mice, *n* = 3, *P* > 0.05. (H) Average long spermatids ratio in adult wild-type and *Tex33*^−∕−^ mice, *n* = 3, *P* > 0.05.

10.7717/peerj.9629/supp-21 Figure S3 Normal semen quality in *Tex33*^+∕−^ mouse (A) Average ratio (%) of motile sperm and (B) progressive sperm from cauda epididymal sperm of adult wild-type and *Tex33* ^+∕−^ mice, *n* = 3, *P* > 0.05. (C) Normal epididymal sperm concentration from adult wild-type and *Tex33* ^+∕−^ mice, *n* = 3, *P* > 0.05.

## Additional Information and Declarations

### Data Availability

The following information was supplied regarding data availability: The raw measurements are available in Tables S1–S18.
