# Supplementary figures and images for: Spermatogenesis is normal in Tex33 knockout mice
Source: PeerJ. 2020 Jul 29;8:e9629. doi: 10.7717/peerj.9629 (PMC7395601; doi:10.7717/peerj.9629)

# Supplementary Figure 1

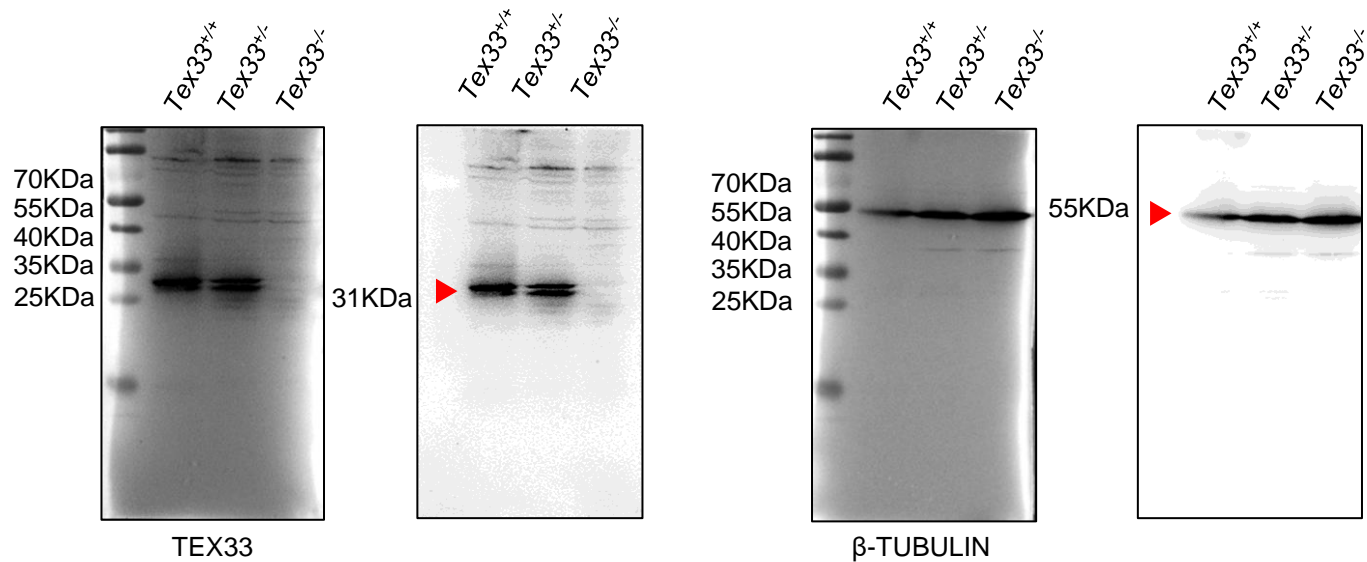

Supplement: Figure S1 — TEX33 is detected in Tex33+∕+ and Tex33+∕− adult male testis, and beta-Tubulin is set as internal control [file peerj-08-9629-s019.pdf]

# Supplementary Figure 2

**A** *Tex33*<sup>+/+</sup> *Tex33*<sup>+/-</sup>

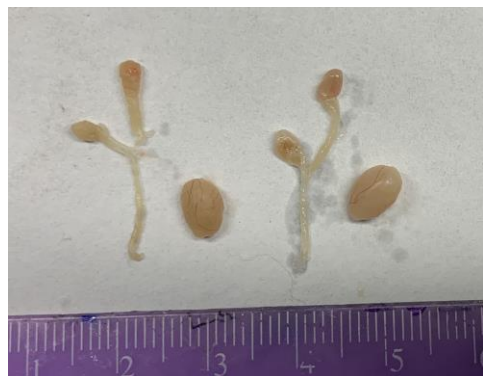

*Tex33*<sup>+/+</sup>

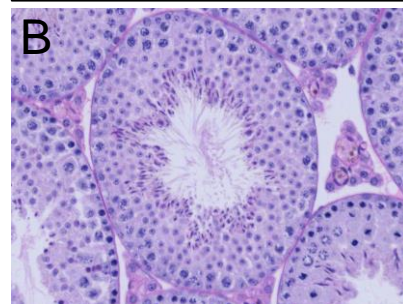

*Tex33*<sup>+/-</sup>

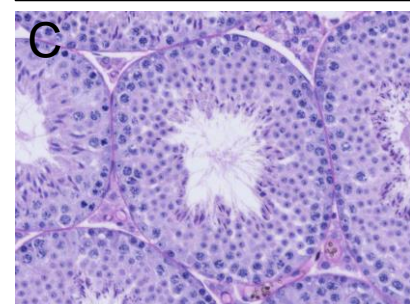

**D**

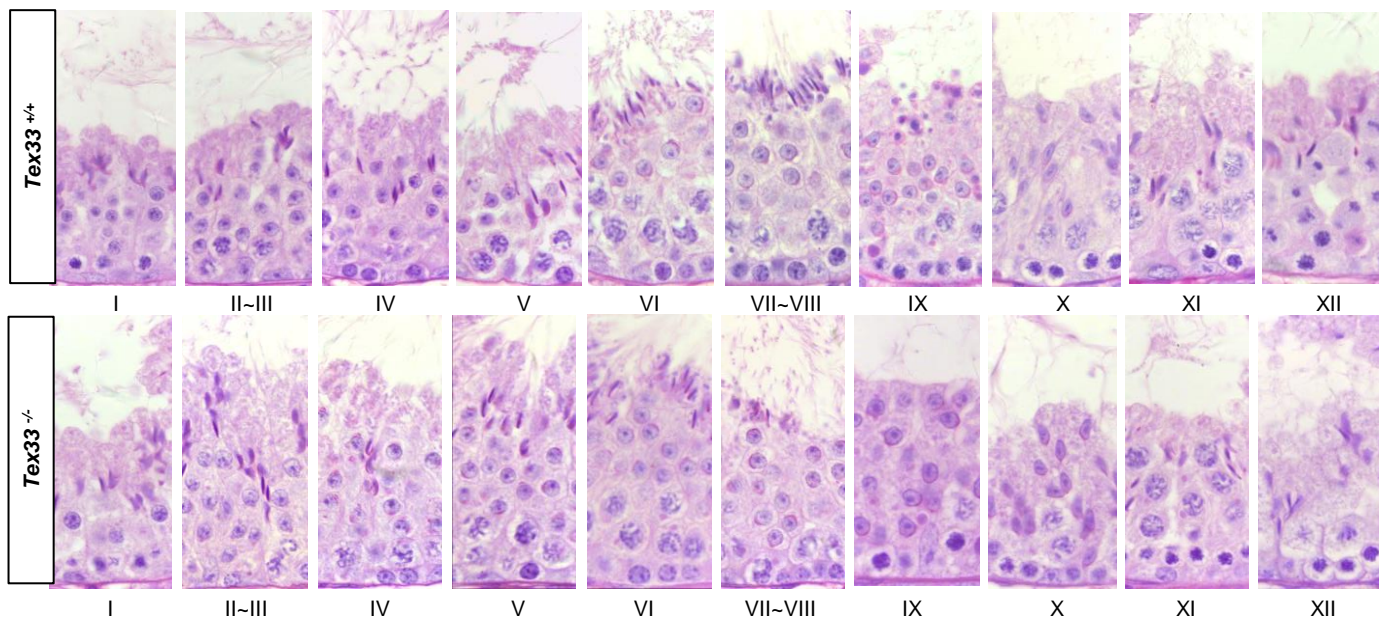

**E**

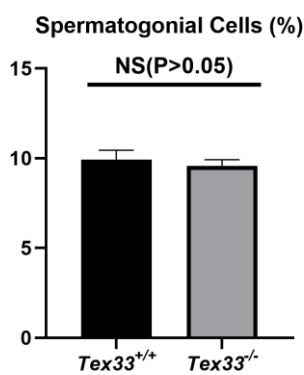

**F**

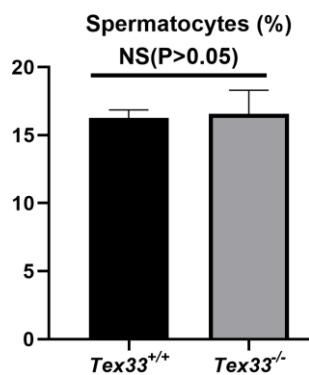

**G**

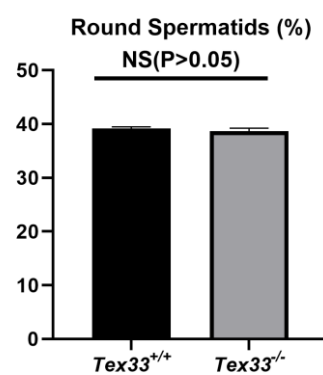

**H**

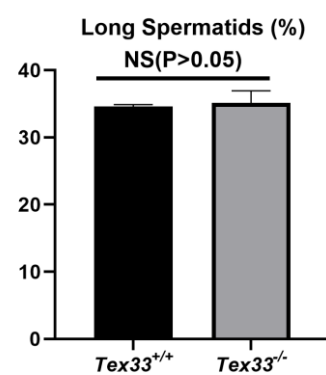

Supplement: Figure S20 — (A) Testis and epididymis from adult wild-type and Tex33+∕− adult mice. Sections of periodic acid Schiff-stained testis from adult (B) wild-type and (C) Tex33+∕− mice; (D) Compared with the wild-type mouse, the adult Tex33−∕− mouse have no spermatogenic abruption and failure in Sections of periodic acid Schiff-stained testis. (E) Average Spermatogonia cells ratio in adult wild-type and Tex33−∕− mice, n = 3, P > 0.05. (F) Average Spermatocytes ratio in adult wild-type and Tex33−∕− mice, n = 3, P > 0.05. (G) Average round spermatids ratio in adult wild-type and Tex33−∕− mice, n = 3, P > 0.05. (H) Average long spermatids ratio in adult wild-type and Tex33−∕− mice, n = 3, P > 0.05. [file peerj-08-9629-s020.pdf]

## Supplementary Figure 3

A

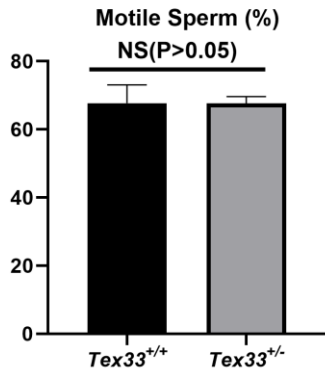

B

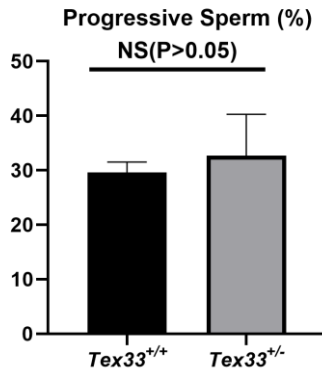

C

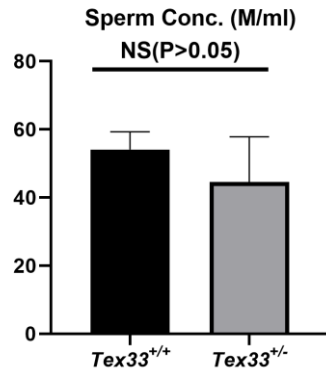

Supplement: Figure S3 — (A) Average ratio (%) of motile sperm and (B) progressive sperm from cauda epididymal sperm of adult wild-type and Tex33 +∕− mice, n = 3, P > 0.05. (C) Normal epididymal sperm concentration from adult wild-type and Tex33 +∕− mice, n = 3, P > 0.05. [file peerj-08-9629-s021.pdf]
